# Tin-filtered 100 kV ultra-low-dose CT of the paranasal sinus: Initial clinical results

**DOI:** 10.1371/journal.pone.0216295

**Published:** 2019-05-06

**Authors:** Bernhard Petritsch, Aleksander Kosmala, Andreas Max Weng, Thorsten Alexander Bley

**Affiliations:** Department of Diagnostic and Interventional Radiology, University Hospital Würzburg, Würzburg, Bavaria, Germany; Chongqing University, CHINA

## Abstract

**Objectives:**

To investigate the feasibility, diagnostic image quality and radiation dose of 3^rd^ generation dual-source computed tomography (CT) using a tin-filtered 100 kV protocol in patients with suspected acute inflammatory sinus disease.

**Methods:**

We retrospectively evaluated 109 consecutive patients who underwent CT (Siemens SOMATOM Force, Erlangen, Germany) of the paranasal sinus with a new tin-filtered scan-protocol (Sn100 kV; tube current 35 mAs) using iterative reconstruction. Two readers independently assessed subjective image quality using a five-point Likert scale (1 = excellent, 5 = non-diagnostic). Inter-observer agreement was calculated and expressed as percentage of agreement. Noise was determined for calculation of signal-to-noise-ratio (SNR). Effective radiation dose (ED) was calculated from the dose-length-product (DLP).

**Results:**

All examinations showed diagnostic image quality regarding evaluation of inflammatory sinus disease. On average, subjective general image quality was rated moderate (= 3) with a percentage of agreement between the observers of 81%. The mean image noise was 14.3 HU. The calculated median SNR was 6.0 for intraorbital fat, and 3.6 for the vitreous body, respectively. The median DLP was 2.1 mGy*cm, resulting in a median ED of 0.012 mSv.

**Conclusions:**

Taking the study limitations into account, ultra-low-dose tin-filtered CT of the paranasal sinus at a tube voltage of 100 kV utilizing an iterative reconstruction algorithm provides for reliable exclusion of suspected acute inflammatory sinus disease in 100% of the cases.

## Introduction

Non-enhanced computed tomography (CT) of the paranasal sinus and facial skull represents the gold standard for a wide spectrum of indications. Especially the extent and severity of inflammatory sinus disease can be determined, thus adding valuable information to the clinical diagnosis of rhinosinusitis [1–5]. Important information regarding bones, surgically relevant anatomical variants and soft tissue can be provided with high spatial resolution and contrast [6–9]. Radiation exposure in this examination has always been a major concern, as radiosensitive organs as the optic lenses (with their vulnerability to radiation-induced cataract development) are included in the scan volume, and are exposed to direct or scattered radiation [3,10–13]. The predominantly young patient population and the potential need for repetitive examinations in the same patient for mostly benign diseases demands strict compliance to the established ALARA (“as low as reasonably achievable”) principle. Various dose-reduction strategies have been discussed, including low-dose protocols based on reduction of tube-current and / or tube-voltage, high-pitch scanning modes, spectral shaping, as well as the use of iterative reconstructions (IR), the latter recently also using novel artificial neural networks (e.g. k-sparse auto encoder; residual encoder-decoder convolutional neural network–RED-CNN) with promising results in terms of noise suppression and structural preservation [3,4,6,14–19]. Although these techniques have effected a considerable dose reduction enabling CT-scans of the paranasal sinus with a radiation dose of not more than 0.019 mSv, further dose-saving strategies are warranted [14]. With the recent introduction of latest (3^rd^) generation dual-source CT-scanners a new powerful x-ray tube (Vectron, Siemens Healthcare GmbH, Erlangen, Germany), which can be coupled with a special tin (_50_Sn) filter, has become available. The tin-filter absorbs low-energy photons that are less relevant in a high-contrast setting (e.g. bone imaging) and, therefore, helps to reduce the radiation exposure of the patient. Along with a tube-voltage of 100 kV, reduced tube-current and the use of iterative reconstruction this setting poses a promising new perspective in low-dose imaging of the paranasal sinus. The positive effects of tin-filtration on image quality and radiation dose in parasinus CT have been shown in cadaver studies [17] and, using a high tube current of 200 mAs, in a small patient population as well [16]. However, all of these former studies did not focus on the special indication of inflammatory sinus disease, which commonly does not require radiation exposure as high as in e.g. trauma imaging.

Thus, the purpose of this retrospective study was to assess the feasibility and image quality of tin-filtered 100 kV CT of the paranasal sinus with comparatively low tube-current of 35 mAs for detection of acute inflammatory sinus disease with special regard to the patient’s radiation dose.

## Materials and methods

The institutional review board (University Hospital Würzburg) approved this study. Consent was not obtained (data were obtained as clinical routine examinations and then retrospectively analyzed anonymously). Image sets of 109 consecutive patients (77 males, 32 females), referred for CT of the paranasal sinus between December 2016 and January 2018 to rule out acute sinusitis were evaluated.

### CT examination

All CT examinations were performed using a third-generation dual-source scanner (SOMATOM Force, Siemens Healthcare GmbH, Erlangen, Germany). Tube voltage was set to Sn100 kV, where Sn indicates the use of a 0.64 mm tin filter; tube current was set to 35 mAs (default settings of the vendor). Automatic anatomical tube current modulation (CARE Dose 4D, Siemens) and automated tube potential control (CARE kV, Siemens) were turned off. Detailed CT acquisition parameters are summarized in Table 1. Patients were scanned in supine position with the head slightly reclined to obtain an almost parallel alignment of the upper jaw to the gantry to minimize impairment by artifacts caused by dental prosthesis. The scan range extended from the roof of the frontal sinus down to the maxilla. The scan direction was caudocranial. By default, 3 mm axial images using a bony (HR40) and soft-tissue (HR36) iterative reconstruction kernel (ADMIRE, Siemens, IR strength level 3 [bony] and 4 [soft-tissue], respectively) were reconstructed from the raw data set [17]. Additionally 3 mm coronal multiplanar reformations (MPR) were generated using the same bony kernel.

**Table 1:** Tin-filtered CT protocol.

Acquisition parameters	
CT system	Siemens SOMATOM Force
CT mode	Single-source / Single-energy
Scan direction	caudocranial
Collimation	192x0.6 mm
Rotation time [s]	0.5 s
Pitch	1.2
Automatic tube current modulation	Off
(CARE Dose 4D, Siemens)	
Automatic tube potential control	Off
(CARE kV, Siemens)	
Tube potential [kV]	Sn 100 kV
Tube current time product [mAs]	35 mAs

### Assessment of image quality parameters

For analysis of subjective image quality all datasets were independently evaluated by two radiologists, with 8 and 4 years of experience, respectively. Images were evaluated using a five-point Likert scale (1 = excellent image quality; 2 = good; 3 = moderate; 4 = fair; 5 = non-diagnostic). The general image impression was analyzed regarding image-noise and the differentiability of common anatomical landmarks usually evaluated within a CT of the paranasal sinus [14,20]. For a more detailed and systematic evaluation the following four bony and soft tissue structures were separately evaluated: the ostiomeatal complex; the septal branches of the ethmoidal air cells; the distinction of the individual mastoid cells in the temporal bone; the orbital cavity with special regard to the differentiation of the eyeball, optic nerve and eye muscles. In addition subjective image quality was evaluated using a binary classification by summarizing scores 1–4 as “diagnostic” *vs*. the score of 5 as “non-diagnostic”. The presence of acute sinusitis was documented for the presence of advanced mucosal swelling and / or the presence of sinusoidal fluid levels by both readers independently using a binary classification (0 = no sinusitis; 1 = sinusitis).

For assessment of objective image quality, the CT attenuation of the intraorbital fat and of the vitreous body (in Hounsfield units [HU]) was measured by one reader using a circular region-of-interest (ROI). To account for anatomic differences between patients the ROI was chosen as large as possible, while carefully avoiding adjacent structures and image artifact in order to prevent partial volume effect. Noise (N) was determined as the standard deviation of air measured anteriorly to the face on the level of the maxillary sinus using a 20 mm ROI. The measurement was repeated twice (ROI placed in front of the right and left maxillary sinus), and the mean result was used for further calculations. Based on these measurements, signal-to-noise ratio (SNR) was calculated as following: SNR = attenuation / [(N of air measured anteriorly to the right face + N of air measured anteriorly of the left face) x 0.5].

### Radiation dose estimates

The estimation of the effective radiation dose (ED) was based on the volume computed tomography dose index (CTDIvol) and dose length product (DLP) which were recorded from the patient protocol, which is automatically stored in the picture archiving and communication system (PACS) (Merlin, Phönix-PACS, Freiburg, Germany). A previously published conversion factor for the head region (0.0019 mSv x mGy^-1^ x cm^-1^; referenced to the 16-cm CTDI-phantom) was used for estimation of the ED [21]. However, due to vendor-specific default settings that apply to all tin-filtered protocols, the Sn100 kV scan is routinely referenced to a 32-cm CTDI-phantom by the scanner. To compensate for the different reference phantom of the tin-filtered protocol a previously published additional conversion factor of 2.5 was applied [17]. Hence, the effective dose results as follows: ED = DLP x 0.0019 mSv x mGy^-1^ x cm^-1^ x 2.5.

### Statistical analysis

Analyses were performed using statistical software (IBM SPSS Statistics for Windows, Version 25.0, Armonk, NY, USA). A *P* value ≤ 0.05 was considered to indicate a statistically significant difference. Results are expressed as median (quartiles) for non-normally distributed variables (DLP, effective radiation dose, SNR) and means ± standard deviations for normally distributed variables (noise). The inter-observer agreement was investigated by calculating the percentage of agreement rate. The reliability for quality assessment of ultra-low-dose CT images and frequency of acute sinusitis was calculated with Cohen’s weighted kappa statistics [22].

## Results

**Fig 1:**
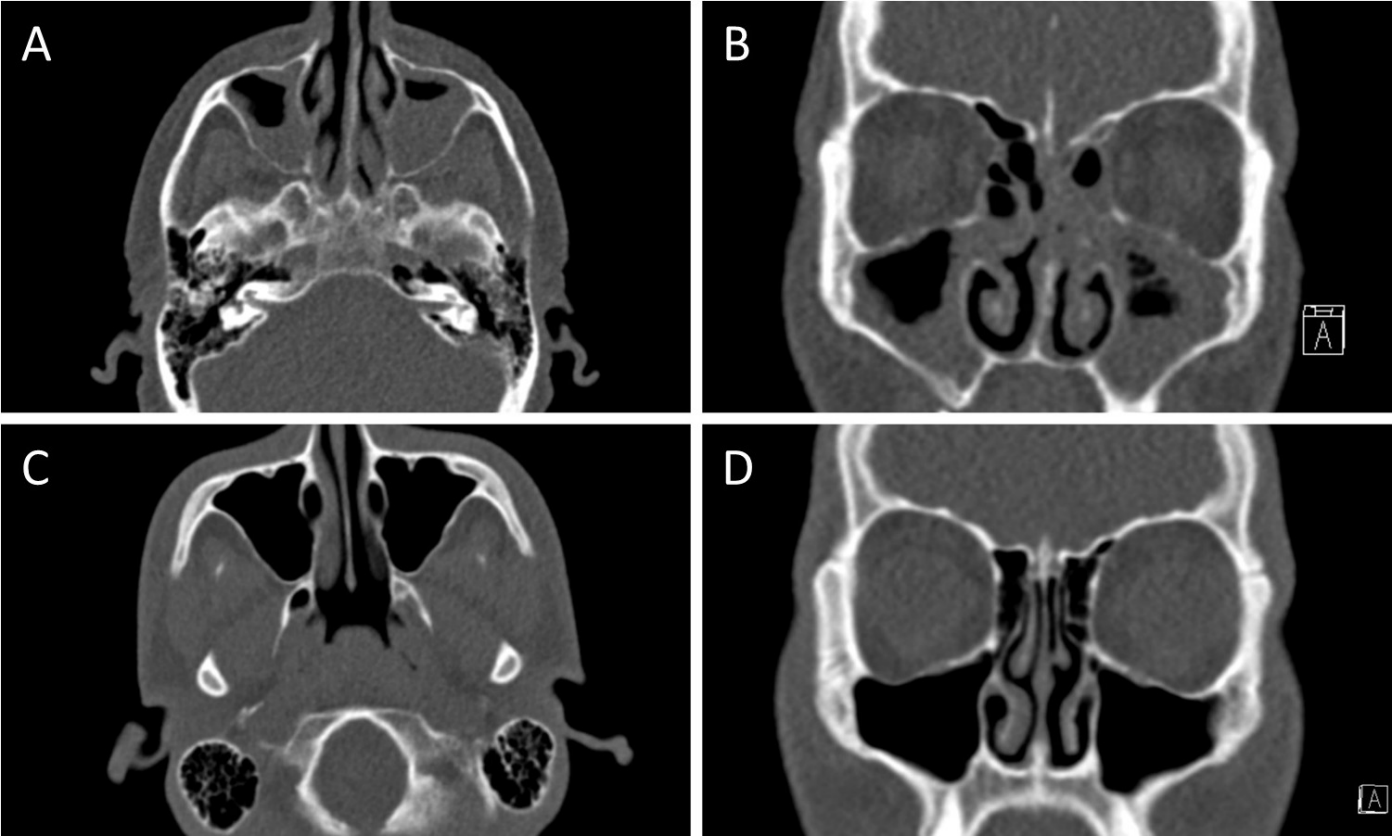
Ultra-low-dose CT-images of paranasal sinus. Axial (A) and coronal (B) reformations of 100 kV tin-filtered ultra-low-dose CT of the paranasal sinus of a 63-year-old male (DLP 2.1 mGycm; effective dose 0.012 mSv). Despite the very low dose, the diagnosis of florid sinusitis is easily established. In a 19-year-old male (axial C, coronal D), sinusitis was sufficiently excluded (DLP 1.8 mGycm; effective dose 0.010 mSv). The general image quality in both patients was rated as “moderate” by both readers.

The mean age of all examined patients was 60 ± 18 years (range 18–93 years). Acute sinusitis was rated present in 25/109 patients by reader 1 and in 23/109 patients by reader 2. The overall inter-observer agreement regarding the diagnosis of sinusitis was excellent (98% agreement) with excellent reliability (ĸ = 0.95; 95% CI 0.87–1.0). A consensus reading was only required in 2 patients who then were classified as sinusitis positive. In the consensus results 25 out of 109 patients (22.9%) were diagnosed as sinusitis positive (Fig 1).

### Radiation dose estimates

The CTDIvol of the applied protocol was 0.12 mGy. The median DLP was 2.1 (2.0; 2.3) mGy*cm [range 1.7–3.8 mGy*cm]. The resulting median effective radiation dose was 0.012 (0.012; 0.013) mSv [range 0.010–0.022 mSv].

### Subjective image quality

The percentage of agreement for general image quality was moderate (81% agreement) with moderate reliability (ĸ = 0.63; 95% CI 0.48–0.77). Details concerning the percentage of agreement of the individual anatomical structures are given in Table 2. Summarizing scores 1–4 as “diagnostic” and leaving 5 as “non-diagnostic”, the inter-observer agreement rate was improved to 100% with perfect reliability (ĸ = 1.00; 95% CI 1.00–1.00).

**Table 2:** Subjective image quality.

	General IQ		OMC		Ethmoidal air cells		Orbital cavity		Mastoid cells	
Score	Reader 1	Reader 2	Reader 1	Reader 2	Reader 1	Reader 2	Reader 1	Reader 2	Reader 1	Reader 2
1	0	0	0	1	0	0	0	0	0	0
2	46	48	47	44	65	57	2	1	30	33
3	60	59	55	55	42	49	46	47	73	67
4	3	2	7	9	2	3	52	52	6	9
5	0	0	0	0	0	0	9	9	0	0
Agreement	81%		78%		73%		79%		76%	
Binary score	100%		100%		100%		100%		100%	
Reliability	ĸ = 0.63		ĸ = 0.61		ĸ = 0.48		ĸ = 0.64		ĸ = 0.52	

*Note*: Image scores of defined anatomical structures: 1 = excellent; 5 = non-diagnostic. Values regarding prevalence of image quality scores are given as absolute numbers (n / 109). Values of agreement are given as percentage (%). Reliability is given as linear weighted kappa value. IQ = image quality; OMC = ostiomeatal complex.

**Fig 2:**
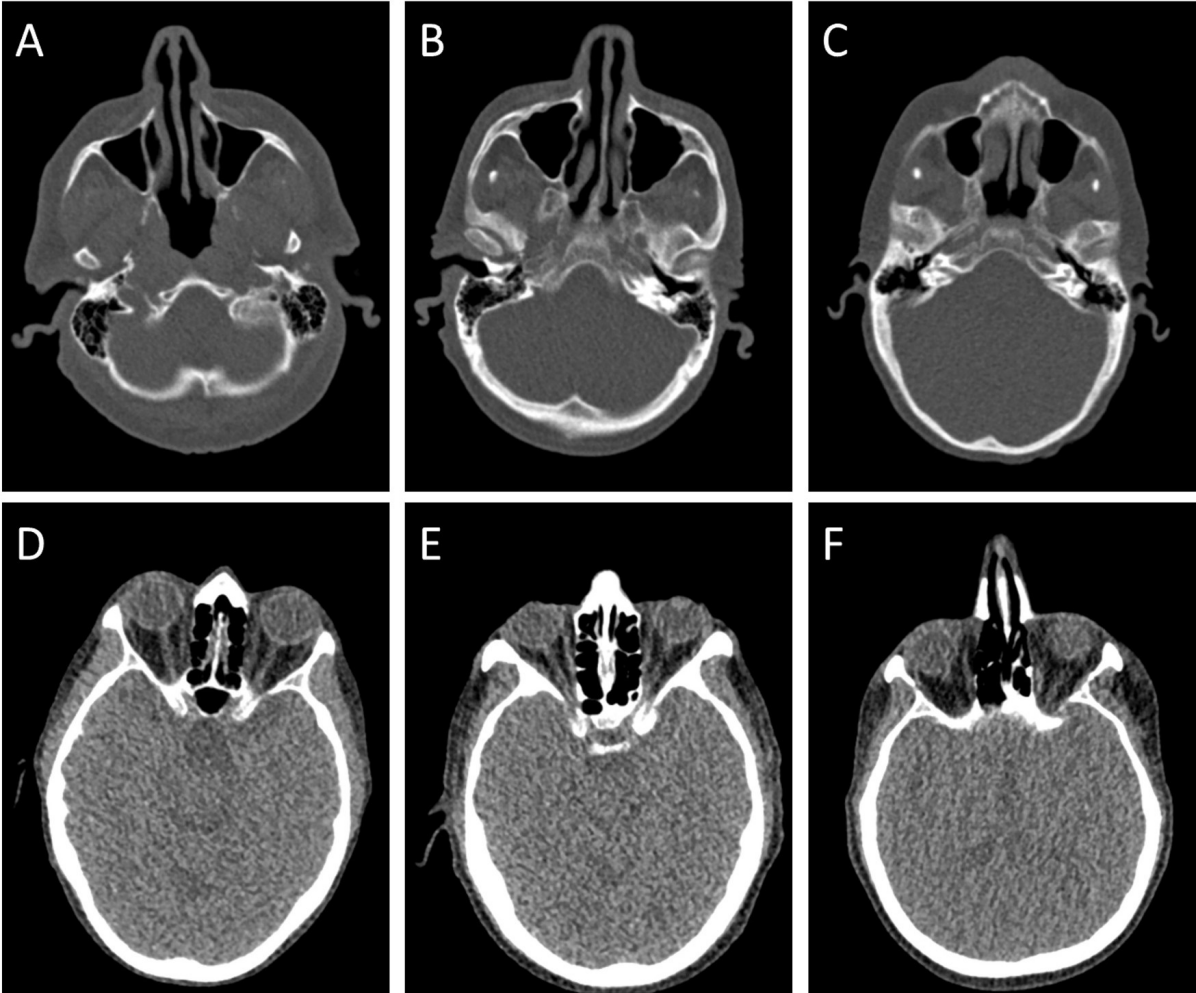
Examples of different image quality scores. Axial bony reformations show good (A), moderate (B) and fair (C) distinction of the individual mastoid cells. Axial soft-tissue reconstructions show moderate (D), fair (E) and non-diagnostic (F) image quality regarding the differentiation of orbital structures.

The general image quality was rated 3 by both readers (Fig 2). Details of the subjective image quality regarding the ostiomeatal complex, the discrimination of ethmoidal cells, the discrimination of structures of the orbital cavity and the distinction of the individual mastoid cells are summarized in Table 2. A reliable evaluation of inflammatory sinus disease was possible in all patients. However, regarding the subgroup analysis of the orbital cavity, a total of nine CT scans were rated non-diagnostic by both readers (score value 5).

### Objective image quality

The mean noise was 14.3 ± 2.5 HU [range 9.4–24.2 HU]. The attenuation of the intraorbital fat was -85.3 ± 9.0 HU [range -66.0 –-111.0 HU] and 51.4 ± 9.3 HU [range 33.0–74.0] for the vitreous body. The calculated median SNR for intraorbital fat was 6.0 (5.2; 6.8) [range 3.4–9.4], and 3.6 (3.0; 4.3) [range 2.1–6.2] for the vitreous body, respectively.

## Discussion

The eye lenses as radiosensitive organs are exposed to direct and scattered radiation during CT of the paranasal sinus [3,10,11,13,14]. Several methods for lens protection during CT-scanning have been described [23,24]. However, the most effective way to minimize radiation dose is the use of a proper lower-dose acquisition technique [4,6,25]. In this study, we demonstrate that the use of a tin-filter in combination with 100 kV tube voltage and a tube current of 35 mAs allows for substantial radiation dose reduction in CT of the paranasal sinus still providing diagnostic image quality of the most important anatomical structures, thus enabling sufficient exclusion of acute sinusitis in 100% of cases.

Previous studies demonstrated promising results with different dose reduction strategies, including high pitch scanning [4,6], low-kilovolt protocols [14], iterative reconstruction technique [3,26], use of neural networks [18,19], spectral shaping [16,17,27], and cone-beam CT [28,29]. Tin-filtration was recently evaluated for imaging of the paranasal sinus in a cadaveric phantom study using different tube voltages and tube currents [17]. The authors of this study suggest that a CTDIvol of 0.2 (Sn100 kV, 25 mAs) could be used to rule out mucosal swelling or fluid retention. A different study addresses the positive effect of tin-filtration at 100 kV yielding sufficient image quality for preoperative planning with an effective dose of < 0.05 mSv [27]. The present study is the first investigation that focuses on minimal radiation dose in suspected acute inflammatory sinus disease. Therefore, more dose-intensive issues as preoperative planning or post trauma imaging could be ignored in the context of the present study.

When Marmolya et al. first introduced the term “*low-dose*” CT relating to the paranasal sinuses in 1991, they specified that 23 mAs at a standard tube-voltage of 120 kV provides sufficient quality for the evaluation of suspected sinusitis [15]. Bulla et al. achieved dose reductions down to a mean DLP of 49.6 mGycm by using iterative reconstructions instead of FBP in a 120 kV (24 mAs) protocol [3]. A similar radiation exposure (mean DLP 51.0 mGycm; ED 0.11 mSv) was reported by Schell et al. who used a high-pitch protocol on a 2^nd^ generation dual-source CT-scanner [6]. In 2012 Schulz et al. introduced the term “*ultra-low-dose*” CT [8] which was taken up Bodelle et al. in 2015 when introducing a 70 kV (75 mAs) protocol, resulting in a mean DLP of 31.1 (ED 0.07 mSv) [14]. A mean DLP of 7.0 mGycm (ED 0.019 mSv) as shown by Aksoy et al. in 2014 is the currently lowest dose reported in literature [4]. With a mean DLP of 2.2 mGycm and a mean ED of 0.012 mSv our proposed ultra-low-dose technique allows for another dose reduction in comparison to previous studies, cutting down the dose to approximately 1/3 to 1/10 of the lowest doses reported in literature so far [2,4,14]. However, the achieved radiation dose in our study is only a minor change to the reported 0.019 mSv by Aksoy et al. [4]. Evidently, the understanding of “*low*” has changed during the past 2 decades. For a more tangible comparison, the annual natural radiation exposure in Germany is 2.1 mSv (resulting in a daily natural ED of 0.0057 mSv.), which is approximately half of the ED dose of our present protocol [30]. Although the ALARA principle is important, the clinical relevance of reducing the radiation dose in such low spheres could be questioned. However, since we are committed to the ALARA principle we find that an exhaustion of the current technical possibilities is reasonable.

Moreover, there are additional benefits of keeping the kV high (e.g. 100 kV) and simultaneously reducing the X-ray flux with additional tin-filtration, compared to the more conventional approach of lowering the kV to reduce the X-ray flux. Higher photon energies can decrease image noise measured in the detector because of reduced absorption in the patient. This issue is especially important in paranasal CT imaging, as due to the high intrinsic contrast between relevant structures–bone *vs*. air, mucosal swelling or fluid–image quality is primarily limited by the image noise and not the image contrast.

Beside detection or rule out of acute inflammatory sinus disease, the present ultra-low-dose protocol might be of special interest when children are assigned for CT imaging of the paranasal sinus. Compared with the European reference dose for CT examination of the face and sinus (DLP 360 mGycm), the 100 kV tin-filter technique with 35 mAs cuts the radiation exposure of the patient down to about 0.6% in the present study [31].

### Study limitations

The present study has several limitations. First, a tin-filter for x-ray prefiltration is not available on the vast majority of CT scanners at the moment. Second, this study only demonstrates the feasibility of a tin-filtered protocol at a specified tube-voltage of 100 kV. The potential for further optimization of image quality and / or radiation dose by tin-filtration at different tube voltages remains unclear and should be evaluated in further studies, which then should also include control-groups. Third, due to the combination of different dose saving strategies (tin-filter / low kV / IR) we cannot declare which of the individual investigations is mainly responsible for the achievement in dose reduction. Fourth, image quality of our proposed protocol was considered to allow a sufficient diagnosis of inflammatory sinus disease. For detailed depiction of anatomic information prior to surgery, in trauma settings, or for planning of 3D surgical navigation the current protocol was not evaluated and might not provide the image quality necessary for these indications. Because of the fact that only one group was evaluated in the current study, the visual score was not based on relatively high-dose CT or standard low-dose CT, respectively. Therefore, theoretically there was a possibility of misdiagnosis in the current study. Last, we used a more up-to-date conversion factor of 0.0019 mSv/mGy*cm for estimation of the effective radiation dose as proposed by Deak et al. in 2010 [21], while the majority of former studies used a conversion factor of 0.0023 mSv/mGy*cm as proposed by the European Working Group for Guidelines on Quality Criteria in CT in 1999 [31]. Furthermore, the present conversion factor has been used to estimate effective radiation dose derived from unfiltered standard 64 slice CT and has therefore not formally been validated in the present context of spectral CT.

## Conclusions

Taking the study limitations into account, ultra-low-dose tin-filtered CT of the paranasal sinus at a tube voltage of 100 kV utilizing an iterative reconstruction algorithm provides for reliable exclusion of suspected acute inflammatory sinus disease in 100% of the cases at a mean effective dose of 0.012 mSv per scan.
